# Effect of Cognitive-Behavioral Therapy on Nocturnal Autonomic Activity in Patients with Fibromyalgia: A Preliminary Study

**DOI:** 10.3390/brainsci12070947

**Published:** 2022-07-20

**Authors:** Germán Prados, Elena Miró, M. Pilar Martínez, Ana I. Sánchez, Vincent Pichot, Marta Medina-Casado, Florian Chouchou

**Affiliations:** 1 Department of Nursing, Faculty of Health Sciences, University of Granada, 18016 Granada, Spain; germanprados@ugr.es; 2 Instituto de Investigación Biosanitaria ibs.GRANADA, 18012 Granada, Spain; 3 Department of Personality, Assessment and Psychological Treatment, Faculty of Psychology, University of Granada, 18071 Granada, Spain; mnarvaez@ugr.es (M.P.M.); aisabel@ugr.es (A.I.S.); 4 Mind, Brain and Behavior Research Center (CIMCYC), University of Granada, 18071 Granada, Spain; 5 INSERM, U1059, Sainbiose, Dysfonction Vasculaire et Hémostase, Université de Lyon, Université Jean Monnet, 42023 Saint-Etienne, France; vincent.pichot@univ-st-etienne.fr; 6 Blood Transfusion Centre and Tissue Bank of Granada-Almería, 18014 Granada, Spain; marta.medina.casado@gmail.com; 7 IRISSE Laboratory (EA4075), UFR SHE, University of La Réunion, 97430 Le Tampon, France; florianchouchou@gmail.com

**Keywords:** fibromyalgia, heart rate variability, insomnia, psychological treatment

## Abstract

Objective: fibromyalgia is a complex chronic pain syndrome characterized by widespread musculoskeletal pain, insomnia and autonomic alterations. Cognitive-behavioral therapy (CBT) is regarded as a promising treatment in fibromyalgia, but its impact on autonomic function remains uncertain. In this research, we studied the effect of CBT on autonomic functions in fibromyalgia. Methods: Twenty-five participants underwent overnight polysomnographic recordings before and after CBT programs focused on pain (CBT-P) or a hybrid modality focused on pain and insomnia (CBT-C). Sleep quality, daily pain, depression and anxiety were assessed by self-reported questionnaires. We analyzed heart rate variability (HRV) using high-frequency power (HF) as a marker for parasympathetic activity, and low-frequency power (LF) and the LF/HF ratio as relative sympathetic markers during wakefulness and at each sleep stage. Results: After treatment, 14 patients (/25, 58.0%) reported improvement in their sleep: 6 in the CBT-P condition (/12, 50%), and 8 in the CBT-C condition (/13, 61.5%). We found that, regardless of the type of CBT, patients who reported improvement in sleep quality (*n* = 14, 58%) had an increase in HF during stages N2 (*p <* 0.05) and N3 (*p <* 0.05). These changes were related to improvement in sleep quality (N2, *r* = −0.43, *p* = 0.033) but not to pain, depression or anxiety. Conclusions: This study showed an improvement in parasympathetic cardiac control during non-rapid-eye-movement sleep following CBT in fibromyalgia participants who reported better sleep after this therapy. CBT may have a cardio-protective effect and HRV could be used as a sleep monitoring tool in fibromyalgia.

## 1. Introduction

Fibromyalgia (FM) is a chronic widespread musculoskeletal pain syndrome with fatigue, sleep disturbances, emotional impairment and cognitive complaints [1]. Although its etiology remains elusive, it is hypothesized that interplay between various mechanisms—including genetic predisposition, stressful life events, and peripheral (inflammatory) and central sensitization mechanisms—leads to pain dysperception [2]. Furthermore, it is well established that psychological (cognitive and emotional) factors play a role in the perception of pain [3,4] and associated disability [5,6]. Autonomic nervous system (ANS) dysfunctions have also been reported, such as predominant sympathetic activity and a decrease in parasympathetic activity, associated with blunted stress reactivity, showing a limited capacity for autonomic cardiovascular adjustment [7,8]. These alterations in parasympathetic control and sympathetic reactivity have been proposed to contribute to the pathophysiology of fibromyalgia, and are related to some of its physical and psychological symptoms [7,8,9]. 

In the last three decades, analysis of heart rate variability (HRV), which is the variability in successive heartbeats, has been used as an indirect measure of autonomic dysregulations in FM [7,10,11,12]. In frequency-domain analyses, and compared to healthy controls, FM patients showed lower total and high-frequency (HF) powers, indicating alterations in cardiac parasympathetic regulation [10,13]. By contrast, increases in the low-frequency to high-frequency ratio (LF/HF ratio)—and sometimes in low-frequency power (LF)—have been shown, illustrating sympathetic predominance in cardiac control in FM patients [9,10,12,13]. However, LF is considered to be determined by the parasympathetic nervous system, and LF and the LF/HF ratio are limited markers of cardiac sympathetic activity [14,15,16]. Consistently, Furlan, et al. 2005 [17], showed, with microneurography—the reference method for sympathetic activity in humans—that FM patients exhibited higher values of muscle sympathetic nerve activity (MSNA) associated with normalized LF, LF/HF and lower HF in comparison with controls. This autonomic imbalance was found at rest, but many studies showed reduced sympathetic reactivity to stress in this clinical population [7,8,14,15,16].

HRV parameters reflecting sympathetic overactivity and alterations in parasympathetic regulation have been related to increased pain perception [9,18,19], cardiovascular risk [11,20], impaired emotional status [9,10,21] and sleep disturbances [22,23] in FM. 

Due to the presence of nightly sympathetic predominance and reduced cardiac parasympathetic modulation [23,24], high cortical activation during sleep [25], sleep disturbances, cognitive dysfunctions in sleep perception [26,27] and emotional distress [28], the sleep problems of FM patients can be understood according to the hyperarousal model of insomnia [29,30]. In line with this model, a chronic psychophysiological activation has been observed in individuals with primary insomnia [31,32,33]. In this regard, it is worth noting that a high proportion of FM patients complain of unrefreshing sleep, difficulties in falling or staying asleep and early morning awakenings [34]. In this context, cognitive-behavioral therapy focused on insomnia (CBT-I), delivered on these patients, is demonstrating short- and long-term improvements in sleep, along with improvements in pain and depressive symptoms [35,36,37]. Cognitive-behavioral therapy focused on pain (CBT-P) is the most common modality applied in this population, and mainly targets disability, pain and related psychological factors [38]; there is evidence that it also leads to clinical improvements in sleep and insomnia symptoms, although to a lesser degree than CBT-I does [35,38,39]. The coexistence of sleep disturbances and chronic pain in multiple chronic illnesses, and the existence of cognitive-behavioral commonalities in the relationship between these phenomena, has led to the emergence of hybrid CBT approaches to simultaneously address them. Despite the very few studies conducted in clinical populations, evidence to date has shown that the hybrid treatment approach has clear potential for enhancing CBT for patients with chronic pain suffering from insomnia [40]. As far as we know, only two clinical trials have been conducted among the FM population, delivering this type of CBT focused on sleep and pain (CBT-C). Concerning sleep benefits, Prados, et al. (2020) [41], found that CBT-C outperformed CBT-P regarding polysomnographic parameters related to refreshing sleep (i.e., higher sleep efficiency, less time awake and longer time in deep sleep) and perceived sleep quality. In the same vein, Lami et al. (2008) [39], found that CBT-C produced better benefits in perceived sleep quality as compared to CBT-P or the usual medical care. In addition, in the study by Lami, patients in the CBT-C condition showed significant gains in pain-related variables, similar to those observed in the CBT-P group. In fact, FM patients who received CBT-C exhibited the best clinical response pattern overall. 

Against the background set out above, FM patients show a high prevalence of cardiovascular risk [20], along with sleep disturbances and chronic pain [42]. The promising new hybrid modalities of CBT being developed might better address the transdiagnostic processes maintaining this illness [43]. Thus, they may enhance the efficiency of this type of psychological treatment, which has traditionally been applied focusing exclusively on pain or insomnia among FM patients [38,44]. Considering this, the objective of this clinical trial was to explore the potential benefits in autonomic regulation after receiving one modality of CBT. Based on previous studies [45,46], we hypothesized that cardiac autonomic control during sleep would be improved following successful CBT-P/CBT-C, with regard to sleep quality improvement.

## 2. Methods

### 2.1. Population

This study is based on secondary analyses of data extracted from a clinical trial that examined the efficacy of two CBT modalities: CBT-P and CBT-C among women with FM suffering from insomnia. The methodology and results regarding the primary research hypotheses are described in Prados, et al. (2020) [41]. A randomized controlled trial was performed using a between-subject experimental design with repeated measures. Women with FM who had received health care in the Rheumatology Service and Pain Unit of Virgen de las Nieves University Hospital, or who belonged to AGRAFIM (a local FM association)—both in Granada, Spain—were screened for eligibility via a brief telephone survey conducted by a psychologist. Next, eligible participants underwent a medical examination by a rheumatologist, and a psychological assessment, based on the following inclusion criteria: (1) having been diagnosed with FM according to the American College of Rheumatology criteria, which were applied by a rheumatologist [47]; (2) having significant self-reported insomnia according to the Diagnostic and Statistical Manual of Mental Disorders (American Psychiatric Association, 2000) [48]; (3) having followed a stable medication regime over the past month. Exclusion criteria were: (1) being pregnant; (2) having a major medical condition; (3) having a severe psychopathology [48]; (4) suffering from other sleep disorders that better explained the insomnia; (5) having a severe dependence on hypnotic drugs; (6) being enrolled in another physical or psychological treatment during the study period. FM participants who met the inclusion criteria, and who had undergone the evaluation sessions, were enrolled in a single night of ambulatory polysomnography (PSG) within the next two weeks, preceding therapy. In addition, a set of questionnaires was given, to be completed at home in one week. This study was approved by the Granada University Ethics Committee. All participants gave their signed, informed consent before engaging in the research protocol.

Considering the inclusion criteria in the PSG analysis, the participants were blindly randomized (1:1) to either the CBT-P or the CBT-C group, using a computerized number generator. The sample was composed of 32 participants (15 in the CBT-P group and 17 in the CBT-C group), who completed the treatment and post-therapy assessment in the primary study. Yet, for the present study, the sample comprised 25 participants (12 in the CBT-P group and 13 in the CBT-C group), after eliminating data from 7 FM females (3 and 4, respectively) who presented poor electrocardiogram (ECG) data for performing the HRV analysis. A per-protocol analysis was carried out in the present study. Thus, the analysis was restricted to participants who finished the study and whose ECG data were available for analyses. 

### 2.2. Intervention Protocol

Starting within one or two weeks after the baseline PSG and questionnaire assessment, participants in both therapy groups (CBT-P and CBT-C) received nine weekly sessions of group therapy that lasted approximately 90 min each. CBT-P focused on psychoeducation on pain physiology, physiological deactivation techniques, goal setting, increasing activity level and social skills, problem-solving and coping with dysfunctional beliefs and rumination related to pain, based on the pain-related fear and avoidance model [49]. CBT-C included the main components of CBT-P, as well as others considering Harvey’s cognitive model of insomnia [50] and the evidence-based practice parameters for the psychological treatment of the insomnia [51]. The protocol for delivering CBT, and its contents, are shown in Table 1.

### 2.3. Measurements

Baseline and post-treatment assessments were performed one to two weeks before and after therapy, respectively. For this study, primary outcomes measures included HRV parameters obtained from the PSG electrocardiogram channels, and perceived sleep quality measured by the Pittsburgh Sleep Quality Index (PSQI). Secondary outcomes were Short-form McGill Pain Questionnaire (MPQ) assessments of daily pain, and Hospital Anxiety and Depression Scale (HADS) measurements of anxiety and depression.

#### 2.3.1. Questionnaires

Pittsburgh Sleep Quality Index (PSQI [52]): This instrument comprises 19 items that assess seven dimensions of sleep quality. The scores of these seven components are added to obtain a global score ranging from 0 to 21. Higher scores in this global measure indicate worse sleep quality. The Spanish version of the PSQI has demonstrated robust psychometric properties in the FM population [53]. The PSQI total scoring was used to define treatment responders concerning this variable. 

Short-form McGill Pain Questionnaire (MPQ [54]): This instrument assesses several dimensions of pain experience using 15 sensory and affective verbal pain descriptors, a daily pain index and a visual analogue scale to evaluate pain intensity during the previous week. The daily pain index was used in this study. Several studies have reported the reliability and validity of the Spanish version of the MPQ [55].

Hospital Anxiety and Depression Scale (HADS [56]): This scale assesses anxiety and depression symptoms in general hospital outpatients. It includes 14 items clustered into anxiety and depression dimensions. This instrument has shown reliable psychometric properties, with sensitivity and specificity levels > 70% in the Spanish FM population [57]. 

#### 2.3.2. Polysomnographic Recordings

PSG was recorded and digitized using SomnoScreen TM plus (SomnoMedics, Germany). Six electroencephalogram (EEG) leads (F3-A2, F4-A1, C3-A2, C4-A1, O1-A2, O2-A1), sampled at 128 Hz, were placed according to the International 10–20 System: two electro-oculogram channels, three electromyogram channels (chin and both legs) and one ECG lead connecting two electrodes placed on the torso aligned in parallel to the right shoulder and left hip (sampled at 256 Hz), were used. Respiration was monitored with a thermistor and a nasal cannula (256 Hz), two piezoelectric belts (32 Hz) for chest and abdominal efforts, and one oxygen saturation channel (4 Hz). Two trained researchers, blinded to group allocation, hooked up the portable device and sensors used for the PSG recording. The starts and ends of the recordings were programmed according to each patient’s sleep schedule, to ensure higher ecological validity. Because the primary study followed the criteria of Rechtschaffen and Kales (1968) [58], sleep stages were blindly rescored by an experienced rater, to update the sleep stages scoring according to AASM criteria [59]. We analyzed sleep variables derived from the PSG analyses, including total time in bed (TIB, the period of time between bedtime and awakening in the morning), TST (the period of time between sleep onset and sleep offset, excluding all wakefulness), and sleep efficiency or SE (TST/TIB). The following variables were also analyzed and expressed as a percentage of TST: time spent in stages N1, N2, N3 and rapid-eye-movement (REM) sleep. In addition, wake time was defined as the percentage of time spent awake from bedtime to final wake-up time. 

### 2.4. Heart Rate Variability Analysis

To study autonomic cardiac activity, a continuous beat-to-beat analysis of the ECG was applied to detect the R peaks of the QRS complex, using HRV analysis, a free software [60]. First, RR intervals (RRIs) were automatically extracted from the ECG signal. If necessary, manual correction of artifacts, ectopic beats, or undetected normal R peaks were applied. Next, R peaks that could not be manually corrected (e.g., artifacts, ectopic beats) were interpolated [60].

Spectral analysis was performed by fast Fourier transform, calculated on sets of RRIs during each sleep stage and wakefulness (i.e., before falling asleep) for each subject. Fourier transform was applied to a 2-Hz resampled RRI signal in a time series of 256 consecutive points, while using a 50% overlap window. The means of these spectra were calculated for each subject and each state of vigilance. HRV indices were calculated as recommended by the Task Force of the European Society of Cardiology and the North American Society of Pacing and Electrophysiology (1996) [61]. High-frequency power (HF) of RRIs (0.15–0.40 Hz) was considered as an index of parasympathetic activity related to breathing control; low-frequency power (LF) of RRIs (0.04–0.15 Hz) represented both parasympathetic and sympathetic activities. The LF/HF ratio was used to explore sympathovagal balance [62].

### 2.5. Statistical Analysis

Analyses were performed using the SPSS-20.0 statistical package (SPSS, Inc., Chicago, IL, USA). Probabilities less than 0.05 were used as the level of significance. Differences between both treatment groups in sociodemographic and clinical characteristics and outcome measures at baseline were compared using *t*-tests or Chi-square statistics. 

The effects of each treatment were analyzed by: (1) normalizing the percentage of basal period values to reduce interindividual variability; (2) dividing the population according to sleep quality improvement (i.e., responders and non-responders); patients with a PSQI score lower than 100% (the basal value) were considered as responders. Next, PSG, HRV and psychological parameters were submitted to a two-sided repeated-measures analysis of variance (ANOVA), with one within-subject factor— Time (pre-treatment vs. post-treatment)—and two between-subject factors—Group treatment (CBT-P vs. CBT-C) and Sleep improvement (responders vs. non-responders). 

Additionally, a univariate analysis, using a two-sided Pearson’s test, was carried out, to explore the association between changes in subjective sleep quality (PSQI), daily pain, depression, anxiety and HRV parameters.

## 3. Results

At baseline, the CBT-P and CBT-C groups were statistically similar in all the sociodemographic and clinical variables measured (all *p* > 0.05; see Table 2). The mean age of the FM participants was 49.97 ± 7.91 years (standard deviation), and 92% of participants in both groups were married or cohabited with a partner. The percentage of participants having completed non-compulsory secondary or higher education was 68%. More than half of the participants (60%) had an inactive work situation (i.e., unemployed, temporary disability or permanent disability). Concerning clinical aspects, the mean duration of FM symptoms was 12.27 years ± 8.12 in the total sample, and approximately 50% of the participants were taking a stable dose of antidepressants, hypnotics or analgesics that did not differ before and after the treatment. At baseline, the CBT-P and CBT-C groups did not show any statistically significant differences regarding self-reported variables or PSG variables (all *p* > 0.05, Table 3).

**Figure 1:**
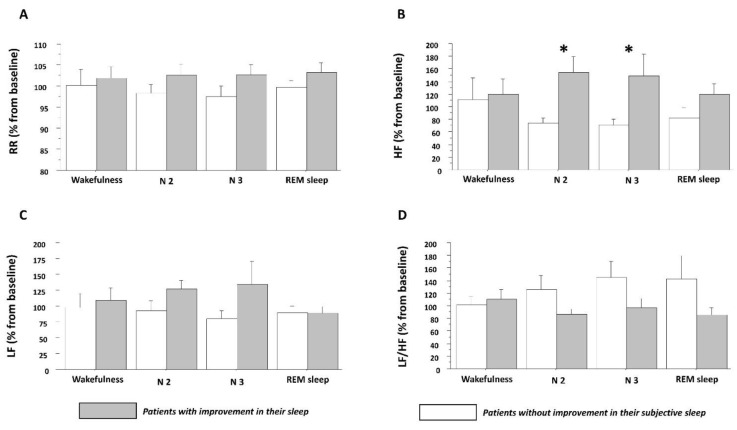
Heart rate variability according to states of vigilance and sleep improvement following CBT. Abbreviations: Cognitive-behavioral therapy (CBT); High-frequency power (HF); Low-frequency power (LF); Ratio of low to high frequency (LF/HF); Stage N2 (N2); Stage N3 (N3); Rapid-eye-movement sleep (REM); RR intervals (RR).

After treatment, 14 patients (/25, 58.0 %) reported improvement in their sleep: 6 in the CBT-P condition (/12, 50%) and 8 in the CBT-C condition (/13, 61.5%). This difference between CBT response was different between groups (*p* < 0.005). As can be seen in Table 4, HRV variables showed a significant effect on Sleep improvement (responders vs. non-responders) in HF power during stages N2 and N3, and a significant effect on Sleep improvement (responders vs. non-responders) × Time (Pre-treatment vs. Post-treatment). Figure 1 shows this significant increase in HF power during stages N2 and N3 following CBT, in patients who reported subjective sleep improvement (decrease in PSQI score, *p <* 0.05) (see Figure 1B), independently of the CBT program they attended (*p* > 0.05). There was no significant change in RRIs, LF/HF or LF power (*p* > 0.05).

Finally, univariate analyses showed that changes in HF power during stage N2 were negatively related to improvement in PSQI score (*p* = 0.033 *r* = −0.43), but this was not observed during stage N3 (*p* = 0.180 *r* = −0.24). As regards psychophysical variables (i.e., daily pain, depression and anxiety reports), we did not find any significant results related to HF power in any sleep stage.

## 4. Discussion

The present study explored the effects of CBT (CBT-P vs. CBT-C) applied to FM patients on autonomic cardiac activity. We observed the following: (1) there was improvement in cardiac parasympathetic control during sleep; (2) these changes in autonomic control of heart rate concerned only patients who reported subjective sleep quality improvement; (3) these changes were observed only during sleep stages N2 and N3; (4) whatever the CBT modality, this psychological treatment improved autonomic regulation of heart rate during non-REM sleep. To our knowledge, this is the first study to explore whether CBT is associated with improvement in nocturnal and diurnal HRV among FM patients with insomnia. As compared to the primary study from which this one is derived, changes observed in self-reported sleep quality in the treatment groups (CBT-P vs. CBT-C) were limited due to the reduction of the sample size imposed by the loss of seven participants with poor ECG data. Nevertheless, and in agreement with the primary study, the percentage of responders concerning perceived sleep quality was higher in the CBT-C (50% vs. 61.5%, respectively) in the present clinical trial [41].

We did not find significant improvement in autonomic cardiac regulation in the whole group of patients after CBT. Nevertheless, patients who reported improvement in perceived sleep (i.e., responders) following any type of CBT, enhanced their cardiac parasympathetic control (increase of HF power) during NREM (N2 and N3) sleep. This positive change was not observed with regard to other HRV parameters in the present study, nor in other sleep stages. We propose that this parasympathetic improvement among responders shows that CBT is a non-pharmacological therapy capable of leading to autonomic cardiac improvement during NREM sleep. These non-REM sleep stages in healthy humans are characterized by parasympathetic predominance and decreased sympathetic modulation, related to both greater baroreflex and respiratory controls and to decreased central modulation in autonomic activity [62]. Considering that ANS regulation during sleep is related to the risk of cardiovascular diseases among people with insomnia [63], FM patients suffering from insomnia could be potential beneficiaries at this health level, after receiving CBT. In addition, promoting parasympathetic cardiac regulation during stages N2 and N3 using CBT may trigger a sleep-protective mechanism and reduce cardiovascular risk [64]. 

Considering studies that have cross-sectionally assessed sleep autonomic profile in FM populations [23,24], CBT-C and CBT-P could be considered as useful interventions to partly reduce the autonomic dysfunctions observed during sleep. In the study carried out by Mork and colleagues [24], FM patients showed lower parasympathetic control of heart rate during stage N2, and a positive correlation was found between HF and sleep quality. In the findings reported by Rizzi and colleagues, parasympathetic dysfunction extended to all the stages of NREM sleep, and there was sympathetic hyper-reactivity during sleep that was compatible with the reduction of vagal modulation during sleep stages [23].

In addition, we can make comparisons between our clinical trial and those that have assessed HRV parameters as outcome results after CBT for insomnia delivered among insomnia patients [45,46]. Firstly, autonomic dysregulations in FM patients have similarities with the pattern observed in insomnia patients: increased sympathetic activation and decreased parasympathetic activity during sleep [23,29]. Secondly, the samples participating in both treatment conditions (CBT-C and CBT-P) in the present study were diagnosed with insomnia after a clinical interview. Thirdly, part of the treatment components delivered in CBT-C was based on the main evidence-based practice parameters for the psychological treatment of insomnia [41]. In contrast with our study, none of the studies referred to above [45,46] found benefits of CBT in autonomic cardiac regulation. Jarrin, et al. (2016) [45], performed a secondary analysis of a clinical trial conducted among patients suffering from insomnia after receiving six weekly group sessions of CBT-I [65]. ECG data obtained pre- and post-treatment from PSG revealed that the improvement in sleep onset latency was related to reduced HF power in sleep stages N2 and REM. These findings were contrary to the expectations of the authors, who had postulated improvements in HRV after therapy. The fact that this trial was carried out in a laboratory was argued by the authors as a possible factor contributing to activation of the stress-response system in participants. An explanation of the benefits found in our study might be the fact that we conducted home ambulatory PSG. Thus, in contrast to Jarrin’s trial, treatment responders in sleep quality were associated with higher HF power in stages N2 and N3 in the present study. 

Johann, et al. (2020) [46], obtained HRV parameters from a 24 h-ambulatory Holter. These authors hypothesized that CBT-I would have benefits on early markers of cardiovascular diseases (i.e., diastolic blood pressure, heart rate, HRV, C-reactive protein, N-terminal pro-brain natriuretic peptide and cystatin C) among patients diagnosed with insomnia, but the results of this clinical trial did not support that idea. An improvement was observed in self-reported measures of insomnia in the CBT-I group, as compared with the waiting list control group, after treatment. Yet, changes in HRV parameters did not show significant effects in daily or nightly recordings for mean 24-h RRIs, LF/HF or SDNN, which is the standard deviation of RRIs and reflects total HRV in time-domain HRV measures. One of the main explanations given by the authors about these results was that the study did not include individuals at increased risk of cardiovascular diseases hampering the potential improvement due to limited floor or ceiling effects. The authors also argued that there was a limited temporal resolution for treatment effects because they had not performed a follow-up, and therefore long-term effects were not assessed. 

Unlike the trials by Jarrin and Johann [45,46], the present one showed significant improvement in parasympathetic control of heart rate in NREM sleep (N2 and N3). Yet, despite our effort to make analogous comparisons between these clinical trials, due to some commonalities regarding the treatments provided and the psychophysiological status of the participants, the results should be interpreted with caution. It should be noted that the participants in our study, besides suffering from insomnia, were diagnosed with FM, as compared to the participants in the other two studies, who were only diagnosed with insomnia. Moreover, the treatment components in the CBT-C in our study were focused on pain and insomnia, and designed for patients with chronic pain. Thus, it is difficult to equate our treatment with those provided in the other clinical trials focused only on insomnia in patients without comorbidities. In addition, there were differences in the treatment dosage in each of the three studies. Specifically, while Johann applied eight weekly individual sessions of CBT-I, in the study by Jarrin and our study, sessions were delivered in groups (six and nine sessions, respectively). Relevant assessment tools also differed between studies that used objective sleep measures (PSG vs. Holter) and those that used subjective measures (PSQI vs. Insomnia Severity Index). In addition, the windows and specificity of analysis of HRV data differed in the study conducted by Johann, which was not able to distinguish between different sleep stages in HRV analyses.

### 4.1. Limitations

Some limitations of the present study are worth noting. Firstly, the sample size was relatively small, reducing the statistical power of our analyses and generalization. Secondly, the lack of follow-up may have hindered the possibility to observe long-term benefits with a wider temporal resolution of the treatment. Thirdly, almost half of the participants in this current study were taking antidepressants, hypnotics or analgesics: these medications could act directly or indirectly on autonomic cardiac regulation [66]. Although we ruled out participants with a severe dependence on psychotropic medications, and it is unrealistic to be able to find a non-medicated FM sample, the use of multiple drugs was an added complication and may have influenced the HRV findings. Fourthly, the two groups differed considerably in educational levels. The small sample size in our study did not make it possible to stratify data in order to analyze plausible associations concerning confounding variables like this one. Fifthly, we included two active treatment conditions in the present study: including a control group that had not received CBT (e.g., a waiting list control group or a group only receiving usual medical care), to control non-specific influences, would have provided relevant information. Sixthly, disability due to pain was not assessed in our sample. Considering that an association between HRV and disability has been reported in other pain conditions [67,68], this assessment would have helped to better describe our sample and to control its impact on the treatment outcome results. Finally, the interpretation of LF and the LF/HF ratio are discussed in the scientific literature [16,69,70]. However, this is not the case for HF, whose interpretation is easier. In fact, this parameter shows significant variations [62]. For all these reasons, these preliminary results need to be confirmed in future broader trials. 

### 4.2. Conclusions

To our knowledge, this is the first study to demonstrate that CBT can improve parasympathetic functions and thus have a cardioprotective effect in patients who suffer from fibromyalgia and insomnia. HRV monitoring appears to be an objective and relevant tool for monitoring sleep improvement in fibromyalgia following CBT. Further studies are needed to confirm these results and explore their underlying mechanisms.

## Figures and Tables

**Table 1:** Components of Cognitive-Behavioral Therapy for Pain and Combined Cognitive-Behavioral Therapy.

	Cognitive-Behavioral Therapy for Pain	Combined Cognitive-Behavioral Therapy
Session 1	Psychoeducation about fibromyalgia and pain psychophysiology.	Psychoeducation about fibromyalgia and pain psychophysiology. Basic information about sleep and factors affecting sleep quality as well as biopsychosocial consequences of sleep loss.
Session 2	Training in physiological deactivation procedures (slow breathing, passive relaxation and imagery training).	Sleep hygiene.
Session 3	Emotion management (raising awareness of the bidirectional relationship between the impact of pain and emotion, training in self-instructions for coping with pain).	Sleep restriction and stimulus control therapy (Sleep diary data were used to calculate sleep efficiency, and to implement a personal sleep schedule for sleep restriction. Participants were trained to review their sleep schedule twice a week, to assess their sleep efficiency, and were supervised by the therapist in a weekly session).
Session 4	Activity pacing and scheduling (managing energy according to rest/activity cycles, setting realistic goals, scheduling pleasant activities).	Training in physiological deactivation procedures (slow breathing, passive relaxation and imagery training).
Session 5	Communication skills training.	Activity pacing and scheduling (managing energy according to rest/activity cycles, setting realistic goals, scheduling pleasant activities).
Session 6	Problem-solving strategies.	Communication skills training and problem-solving strategies.
Session 7	Cognitive therapy: Analyzing relationships between thoughts, feelings and behaviors related to pain. Identifying negative thoughts and dysfunctional beliefs, pain catastrophizing and hypervigilance, and their relationships with activity avoidance and mood state.	Cognitive therapy: Analyzing relationships between thoughts, feelings and behaviors related to pain and sleep. Identifying negative thoughts and dysfunctional beliefs about pain and sleep, pain catastrophizing and hypervigilance, and their relationships with activity avoidance and mood state.
Session 8	Cognitive therapy: Challenging negative thoughts and dysfunctional beliefs about pain (strategies for modifying catastrophic appraisals of pain, and replacing dysfunctional beliefs with more functional ones).	Cognitive therapy: Challenging negative thoughts and dysfunctional beliefs about pain and sleep (strategies for modifying catastrophic appraisals of pain and sleep, and replacing dysfunctional beliefs with more functional ones).
Session 9	Summarizing and remembering key therapy components. Maintaining therapy achievements and preventing relapses.	Summarizing and remembering key therapy components. Maintaining therapy achievements and preventing relapses.

**Table 2:** Demographic and clinical features of fibromyalgia participants.

	Total Sample	CBT-P Group	CBT-C Group	*p* Value
*N*	25	12	13	
Age. *M* (*SD*)	49.97 (7.91)	51.67 (5.33)	47.92 (9.58)	0.23
BMI (kg/m^2^). *M* (*SD*)	27.17 (4.89)	27.44 (2.88)	26.92 (6.33)	0.78
Duration since diagnosis (years). *M* (*SD*)	6.24 (5.19)	5.79 (5.44)	6.70 (5.17)	0.71
Duration of symptoms (years). *M* (*SD*)	12.27 (8.12)	10.91 (7.42)	13.64 (8.91)	0.44
Married/cohabiting (%)	92.0%	100.0%	84.6%	0.16
Non-compulsory secondary or higher education (%)	68.0%	50.0%	84.6%	0.06
Currently employed (%)	40.0%	50.0%	30.8%	0.33
Antidepressants (%)	50.0%	58.3%	41.7%	0.42
Hypnotics (%)	54.2%	66.7%	41.7%	0.22
Anti-inflammatory drugs (%)	33.3%	33.3%	33.3%	0.67
Analgesics (%)	52.2%	50.0%	53.8%	0.85

Abbreviations. CBT-C: cognitive-behavioral therapy for insomnia and pain; CBT-P: cognitive-behavioral therapy for pain. Note: *p* values refer to Chi-square tests for dichotomous variables and *t*-tests for continuous variables.

**Table 3:** Polysomnographic and psychometric data.

	CBT-P (*n* = 12)		CBT-C (*n* = 13)		Comparisons at Baseline
Polysomnography	Baseline [*M* (*SD*)]	Post-Therapy [*M* (*SD*)]	Baseline [*M* (*SD*)]	Post-Therapy [*M* (*SD*)]	*p*
TST (min)	368.90 (51.24)	400.75 (22.03)	351.32 (62.50)	364.27 (53.40)	0.452
SE (%)	85.32 (8.06)	85.85 (6.68)	85.12 (7.94)	91.01 (5.06)	0.950
% Wakefulness	14.64 (8.09)	14.14 (6.69)	14.78 (7.91)	8.99 (5.06)	0.966
% N2	49.33 (8.65)	42.38 (8.91)	48.47 (12.18)	47.74 (6.45)	0.842
% N3	12.95 (8.10)	14.61 (7.75)	15.65 (6.85)	18.70 (5.08)	0.377
% REM	14.82 (6.25)	17.88 (6.83)	14.19 (4.33)	16.99 (6.31)	0.772
Questionnaires					
PSQI-Sleep quality	15.14 (4.56)	15.43 (3.31)	15.42 (4.42)	13.25 (4.94)	0.695
MPQ-Daily pain	2.71 (0.76)	2.71 (0.76)	2.58 (0.51)	2.50 (1.00)	0.523
HADS-Depression	12.00 (3.74)	11.57 (3.74)	8.92 (4.42)	7.75 (4.37)	0.106
HADS-Anxiety	12.43 (5.09)	12.14 (4.81)	11.50 (5.39)	11.58 (5.65)	0.695

Abbreviations. CBT-C: combined cognitive-behavioral therapy; CBT-P: cognitive-behavioral targeting pain; HADS: Hospital Anxiety and Depression Scale; MPQ: McGill Pain Questionnaire; N2: percentage of time in stage N2; N3: percentage of time in stage N3; PSQI: Pittsburgh Sleep Quality Index; REM: rapid-eye-movement sleep; SE: sleep efficiency; TST: total sleep time. Note: *p* values refer to *t*-tests.

**Table 4:** Changes in HRV parameters in the treatment groups according to responders vs. non-responders to perceived sleep quality.

Parameters	State of Vigilance	Time		Type of CBT		Improvement in Sleep		Interaction Time * Type of CBT		Interaction Time * Sleep		Interaction Type of CBT * Sleep		Interaction	
		*t*	*p*	*t*	*p*	*t*	*p*	*t*	*p*	*t*	*p*	*t*	*p*	*t*	*p*
**RRIs**	**wakefulness**	0.1	0.722	1.1	0.3	0.2	0.633	1.1	0.3	0.2	0.633	0.5	0.493	0.5	0.493
	**N2**	0.1	0.938	0.4	0.534	1.28	0.271	0.4	0.534	1.3	0.271	1.8	0.195	1.8	0.195
	**N3**	0,0	0.967	0.5	0.481	2.0	0.173	0.5	0.481	2.0	0.173	1.0	0.33	1.0	0.33
	**REM sleep**	0.6	0.452	1.7	0.207	0.8	0.37	1.7	0.207	0.8	0.37	0.4	0.558	0.4	0.558
**HF**	**wakefulness**	0.6	0.448	2.4	0.14	0.1	0.716	2.4	0.14	0.1	0.716	0.2	0.653	0.2	0.653
	**N2**	0.9	0.343	0.4	0.546	7.7	***0.012***	0.4	0.546	7.7	***0.012***	0,0	0.956	0,0	0.956
	**N3**	0.5	0.476	0.9	0.369	4.8	***0.042***	0.9	0.369	4.8	***0.042***	1.1	0.298	1.1	0.298
	**REM sleep**	0,0	0.965	0.8	0.378	2.2	0.857	0.8	0.378	2.1	0.156	0,0	0.857	0,0	0.857
**LF**	**wakefulness**	0.1	0.73	1.6	0.218	0.3	0.583	1.6	0.218	0.3	0.583	0.1	0.789	0.1	0.789
	**N2**	0.9	0.35	0.2	0.707	2.4	0.135	0.1	0.707	2,0	0.135	0.3	0.594	0.3	0.594
	**N3**	0.4	0.54	0.5	0.49	2.4	0.14	0.4	0.54	2.4	0.14	2.8	0.11	2.8	0.11
	**REM sleep**	1.7	0.197	0.2	0.63	0,0	0.89	0.2	0.63	0,0	0.89	0.4	0.513	0.4	0.513
**LF/HF**	**wakefulness**	0.5	0.507	0.2	0.628	0.1	0.709	0.2	0.628	0.2	0.628	1.5	0.23	1.5	0.23
	**N2**	0.4	0.539	0.6	0.455	3.5	0.076	0.6	0.455	3.5	0.076	0.5	0.497	0.5	0.497
	**N3**	2.5	0.138	2.5	0.128	0.4	0.535	0.4	0.535	2.5	0.128	2.4	0.135	2.4	0.135
	**REM sleep**	0.6	0.44	2.9	0.103	2.3	0.144	0.6	0.446	2.9	0.103	0.6	0.446	0.6	0.446

Abbreviations: cognitive-behavioral therapy (CBT); high-frequency power (HF); low-frequency power (LF); ratio of low to high frequency (LF/HF); RR intervals (RRIs); stage N2 (N2); stage N3 (N3); rapid-eye-movement sleep (REM).

## Data Availability

G.P. and M.P.M. have full access to the data and are the guarantors of the data.
